# Development and Characterization of Polyphenon 60 and Caffeine Microemulsion for Enhanced Antibacterial Activity

**DOI:** 10.1155/2014/932017

**Published:** 2014-06-22

**Authors:** Sonal Gupta, Rakhi Bansal, Javed Ali, Reema Gabrani, Shweta Dang

**Affiliations:** ^1^Department of Biotechnology, Jaypee Institute of Information Technology, A-10, Sector 62, Noida, Uttar Pradesh 201307, India; ^2^Faculty of Pharmacy, Jamia Hamdard, Hamdard Nagar, New Delhi 110062, India

## Abstract

Green tea catechins and caffeine have exhibited antibacterial activity; however, their use is limited by lack of stability and effective delivery systems. Polyphenon 60 (P60) and caffeine were encapsulated in a single microemulsion (ME) formulation with an objective to lower the minimum inhibitory concentrations (MICs) of the individual agents against selected pathogens (*S. epidermidis* and *E. coli*). Combination of two natural compounds would advocate two different mechanisms on the bacterial growth thereby providing for better antibacterial activity. Thermodynamically stable ME was developed and characterized with an average particle size of 17.58 nm, further confirmed by TEM analysis. Antibacterial studies included chequerboard microdilution assay to determine the MIC and fractional inhibitory concentration (FIC) of both the natural compounds individually and in combination. MIC and FIC results indicated that the combination of the above two natural compounds was proficient in lowering the MICs of individual agents. Results of DPPH assay indicated that ME system preserved the long term antioxidative potential of P60 and caffeine. The cytotoxicity of the optimized formulation on Vero cell line by MTT assay was found to be nontoxic to mammalian cells.

## 1. Introduction

Use of antibiotics is referred to as the blockbuster therapy model to treat different types of infections. However, the continuous use of antibiotics might lead to the problem of drug resistance development in the patients [1]. Therefore, the use of plant derived compounds as antimicrobial agents comes into the picture as a safe and effective alternative. Synergistic action of two compounds is well reported by numerous researchers with an objective to lower the MICs of the individual agents that are used in the combination [2]. Therefore, the combination would be safe for human use when used at relatively lower concentrations. Combination of two natural compounds would advocate two different mechanisms on the bacterial growth thereby providing for better antibacterial activity. Moreover, the nonspecific action of natural compounds in combination will not allow bacteria to become resistant [3] unlike antibiotics that cause selective pressure on pathogens thereby switching them to drug resistant strains [4].

Green tea catechins are polyphenolic compounds present in unfermented dried leaves of the plant* Camellia sinensis*. Green tea catechins exhibit several pharmacological effects including antimicrobial activity that can be majorly attributed to one of the catechins epigallocatechin gallate (EGCG) [5]. We have previously reported that green tea prevented the adhesion of pathogen to the mammalian cell line, as the possible mechanism of antibacterial action of the green tea extract [6]. The limited therapeutic potential of green tea catechins is reported due to its poor stability and low bioavailability [7]. Caffeine is a bitter crystalline xanthine alkaloid that is extracted from the seed of the coffee plant and the leaves of the tea bush. Antimicrobial effects of caffeine are also well reported [8] but attention has not been focused intensively to evaluate the combination of green tea catechins (P60) and caffeine against bacterial growth. Recent studies signified the use of MEs as efficient antimicrobial agents. Moreover, MEs have been reported to be self-preserving antimicrobial agents. Al-Adham et al. reported that antibacterial activity of antibiotics encapsulated in MEs could be because of the action of MEs on cell membranes of the bacteria [9].

In the present study we encapsulated P60 and caffeine into a single ME formulation and studied its antibacterial efficiency by determining MIC and FIC values. Long term effect on antioxidant potential of ME was also checked via DPPH assay. Finally, the cytotoxicity analysis of the encapsulated P60 + CAF was carried out on mammalian cell line to validate its likely use in humans.

## 2. Materials and Methods

Labrasol and caffeine were kindly gifted by Gattefosse (India) and Himedia Labs (India), respectively. Polyphenon 60, Cremophor EL, Dulbecco's modified eagle medium, and fetal bovine serum were obtained from Sigma-Aldrich (India). Trolox was purchased from Calbiochem (unit of Merck Millipore, India). Nutrient dehydrated agar and nutrient dehydrated broth were obtained from Qualigens, India. Dimethyl sulphoxide was obtained from CDH, India. All the other chemicals used in the study were of analytical grade or HPLC grade.

### 2.1. Procurement and Maintenance of Bacterial Strains

Bacterial strains,* Staphylococcus epidermidis* (MTCC 435) and* Escherichia coli* (MTCC 739), were obtained from MTCC, Chandigarh, India. Both bacterial cultures were maintained in nutrient broth.

### 2.2. Preparation of Polyphenol 60 and Caffeine Loaded ME (P60 + CAF)

#### 2.2.1. Selection of Excipients

Solubility of P60 and caffeine in various oils (sesame oil, olive oil, clove oil, linseed oil, coconut oil, corn oil, canola oil, labrasol, soybean oil, and almond oil), surfactants (tween 20, span 80, and cremophor EL), and cosurfactants (plurol oleique, capryol 90, transcutol P, glycerol, and isopropanol) was checked. An excess amount of P60 and caffeine were added together in 2 mL of the selected oil, surfactant, and cosurfactant in stoppered vials and then preliminary mixing was carried out over magnetic stirrer for few minutes. Later on, these vials were kept in mechanical bath shaker for 48 h at 37°C and checked for homogeneity.

#### 2.2.2. Construction of Pseudoternary Phase Diagram

Oil-in-water MEs were prepared by aqueous phase titration method. *S*_mix_ ratios were varied from 1 : 0, 1 : 1, 2 : 1, 3 : 1, 4 : 1, 5 : 1, and 6 : 1. For individual *S*_mix_ ratio, different combinations of oil and *S*_mix_ were tried (1 : 9, 1 : 8, 1 : 7, 1 : 6, 1 : 5, 1 : 4, 1 : 3.5, 1 : 3, 1 : 2.3, 1 : 2, 1 : 1.5, 1 : 1, 1 : 0.7, 1 : 0.43, 1 : 0.25, and 1 : 0.1) and pseudoternary phase diagram was plotted to study the area of ME. The prepared MEs were observed for transparency/turbidity, viscosity (flow), and phase separation [10].

### 2.3. Characterization of ME

#### 2.3.1. Thermodynamic Stability of ME

To assess the thermodynamic stability of P60 + CAF loaded ME, clarity and phase separation were evaluated before and after subjecting the ME to heating cooling cycle (six cycles between refrigerator temperature (4°C) and (45°C)), centrifugation (3500 rpm for 30 min), freeze thaw cycle (−21°C and +25°C), and water dispersibility test by gently vortexing 1 mL of ME with 10 mL of water.

#### 2.3.2. Droplet Size and Size Distribution

Droplet size was determined by photon correlation spectroscopy that analyzed the fluctuations in light scattering due to brownian motion of the particles, using a Zetasizer (100 HS, Malvern Instruments, UK). The formulation (0.1 mL) was dispersed in 50 mL of water in a volumetric flask and mixed thoroughly with vigorous shaking and light scattering was monitored at 90° angle. Polydispersity index (PDI) for the formulation was determined [11].

#### 2.3.3. Zeta Potential Measurement

Zeta potential analysis was carried out for the ME and its corresponding placebo (diluted 1 : 50, volumetric ratio) using Zetasizer (100 HS, Malvern Instruments, UK) [12].

#### 2.3.4. Morphology

The morphology of ME was observed under TEM (TECNAI 200 Kv TEM (Fei, Electron Optics, USA)) by using negative staining method. A drop of ME, diluted with water (1 : 50 times), was spread on a 200 mesh copper grid coated with carbon film and kept for about 3 min. A drop of phosphotungstic acid (2%, w/w) was dripped on the grid for 30 s and excess droplet was removed using a filter paper. Finally, the grid was air dried for about 3 h and then used for microscopic analysis [13].

### 2.4. Determination of Antimicrobial Activity

#### 2.4.1. Disc Diffusion Assay

Antimicrobial susceptibility tests were carried out by Kirby Bauer's disc diffusion method. Overnight cultures were reinoculated and grown for 3-4 h till absorbance at 600 nm was in the same range as that of 0.5 McFarland standard (absorbance at 600 nm should be from 0.08 to 0.13 for 1-2 × 10^8^ cfu/mL) [14]. The inoculum was diluted to final concentration of 5 × 10^5^ cfu/mL and plated on nutrient agar. The sterile discs (Whatman Filter Paper) were impregnated with 20 *μ*L of aqueous P60, aqueous Caffeine, P60 loaded ME, Caffeine loaded ME, P60 + CAF loaded ME, and corresponding placebo. Gentamicin (4 *μ*g/mL) was used as the positive control. The discs were applied on agar plates and incubated at 37°C for 16 h and zone of inhibitions was measured [15].

#### 2.4.2. Determination of Minimum Inhibitory Concentration (MIC) and Fractional Inhibitory Concentration (FIC)

Stock solutions of aqueous P60 (6.6 mg/mL) and caffeine (13.3 mg/mL) were prepared and further serially diluted (up to 6 dilutions) in the nutrient broth. 100 *μ*L of each of the dilution was added to 96-well plates containing equal volume of bacterial inoculum (5 × 10^5^ cfu/mL). To account for the effect of P60 colour, absorbance (Abs) was taken at 595 nm using ELISA reader at the beginning of the assay (*t*_0_) and after incubation for 12–16 h at 37°C (*t*_16_). The mean percentage inhibition was used to determine the MIC values and calculated according to the formula (1 − ((Abs. of sample at* t*_16_ − Abs. of sample at* t*_0_)/(Abs. of growth control at* t*_16_ − Abs. of growth control at* t*_0_)) ∗100) [16]. All these experiments were repeated thrice to get the concordant results.

In order to determine the MIC for MEs, different concentrations of P60, Caffeine, and P60 + CAF (MIC_aq_, MIC_aq_/2, MIC_aq_/4 and MIC_aq_/8) were encapsulated in the ME system and then subjected to chequerboard microdilution assay as described above.

FIC for P60 + CAF in both aqueous form and ME formulation were calculated as the MIC of an agent in combination divided by the MIC of that agent alone. FIC_index_ was obtained by adding the FICs of P60 and caffeine. If the FIC index was ≤0.5, the combination was defined as synergy and it is additive if FIC index < 0.5–1 [17].

### 2.5. Antioxidative DPPH Assay

The picrylhydrazyl (DPPH) assay was carried out in a 96-well microtiter plate. 5 mM DPPH reagent was freshly prepared in methanol and its absorbance was adjusted to 0.7 at 517 nm by diluting with methanol [18]. Standard curve was plotted using trolox (0.005–0.05 mM). To 100 *μ*L of DPPH solution, same volume of the test sample, that is, aqueous P60 and its ME, aqueous caffeine and its ME, and aqueous P60 + CAF and its ME at their MIC values, was added in the wells of the microtiter plate. The plates were incubated at 37°C for 20 min and the absorbance of each solution was measured at 490 nm using ELISA reader against the positive control (DPPH only). Percentage inhibition is calculated according to ((Abs. of the control − Abs. of the sample)/Abs. of the control)∗100 [19]. In contemplation of confirming the long term activity of MEs, experiments were repeated after one and two weeks, respectively, using the same test samples as prepared on the first day.

### 2.6. *In Vitro* Cytotoxicity Analysis on Vero Cell Line

Vero cell line was maintained in DMEM medium containing 10% fetal bovine serum. Vero cells (10^5^ cells/mL) were seeded in 96-well plate and incubated at 37°C with 5% CO_2_ for 24 hrs to allow the cells to adhere to the plate. Cells were treated with both aqueous and ME formulations of P60 + CAF at the optimized concentrations (corresponding to their respective MIC values) including placebo. After incubation for 24 hrs, 20 *μ*L of MTT (3-(4, 5-dimethylthiazol-2-yl)-2, 5-diphenyltetrazolium bromide) prepared in D-PBSA (5 mg/mL) was added to each well and again incubated for 4 hrs. The media was replaced by 200 *μ*L of DMSO to terminate the assay [20]. Absorbance was taken at 570 nm using an ELISA plate reader. The viability (%) was calculated according to the formula ((*A*_*f*_/*A*_*c*_)∗100), where *A*_*f*_ is absorbance obtained for cells treated with the formulation and *A*_*c*_ is absorbance obtained for positive control (cells without test formulation).

### 2.7. Statistical Analysis

The whole data in the experiment represent the result of three independent experiments. The data were analysed by one-way analysis of variance (ANOVA) using Analysis ToolPak which is an Excel add-in program. Significant differences of means were determined by Fisher (*F*) test *P* value calculator [21].

## 3. Results and Discussion

### 3.1. Preparation and Characterization of P60 Loaded ME

**Figure 1:**
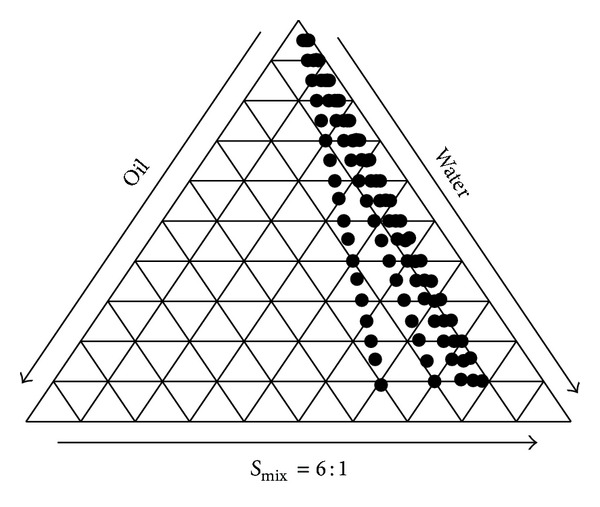
Pseudoternary phase diagram of microemulsion regions of existence (represented by dots) with *S*_mix_ ratio (6 : 1).

P60 + CAF loaded oil-in-water ME was prepared using Labrasol as an oil phase, Cremophor EL as surfactant, and glycerol as cosurfactant. The ternary phase diagram for *S*_mix_ 6 : 1 ratio exhibited maximum transparent region (Figure 1).

The MEs corresponding to this ratio were subjected to thermodynamic stability studies as described above. No phase separation was observed in the ME samples showing good thermal stability.  Table 1 shows the results of particle size analysis, polydispersity index (PDI), and zeta potential of P60 + CAF ME and its placebo. Placebo and P60 + CAF ME had PDI ranging from 0.179 to 0.229, indicating narrow size distribution. The average droplet size of placebo (12.78 nm) was lower compared to P60 + CAF ME (17.73 nm) (Table 1) indicating that the addition of P60 and caffeine increased the droplet size of ME. Therefore, it can be concluded that P60 and caffeine accumulated in the interfacial layers rather than staying in continuous phase (water) [22].

**Figure 2:**
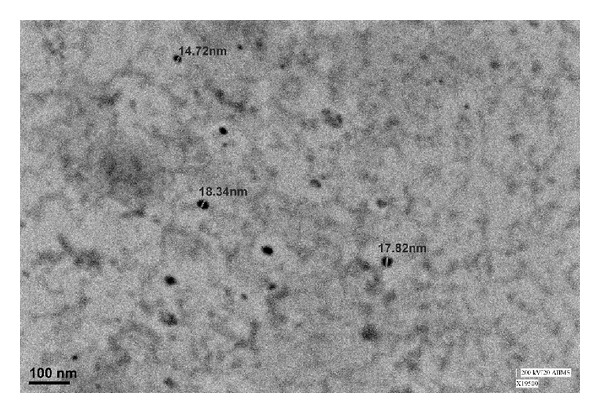
TEM image of P60 + CAF ME.

Despite the nonionic nature of Cremophor EL and glycerol, they are known to decrease the zeta potential measurements [23]. Negative zeta potential of MEs produces steric repulsive forces of hydrocarbon chains which protrude into oil phase thereby hindering aggregation with nearby oil droplets [24]. Therefore, negative zeta potential is imparting stability to the ME system. Zeta potential of P60 + CAF ME and its corresponding placebo have been shown in Table 1.  Figure 2 presents the morphology of P60 + CAF ME, performed by TEM, and further supports the result of zeta size analysis that also indicated the average droplet size ranging from 17.58 nm to 17.96 nm.

### 3.2. Antimicrobial activity

*S. epidermidis* and* E. coli* were screened for sensitivity to the aqueous P60, caffeine, P60 + CAF, their corresponding MEs, and placebo via Kirby-Bauer disc diffusion assay. All the strains were found to be sensitive (zone of inhibition ≥ 7 mm) with larger zone of inhibition obtained for* S. epidermidis* (gram-positive bacteria) compared to* E. coli* (gram-negative bacteria). However, placebo was found to be least active among all the formulations for both the bacterial strains (Table 2). Gentamycin (4 *μ*g/mL) was used as positive control and exhibited an average zone of inhibition of 25 mm.

Higher antimicrobial effect of MEs can be attributed to the formation of nanodrops that increase the surface tension and thereby force themselves to merge with the lipids present in the bacterial cell membrane [25]. On a mass scale, this effectively disintegrates the membrane and kills the bacteria. Moreover, water present in ME system is tightly bound to the internal oil phase and therefore not available to bacteria for its growth [9].

**Figure 3:**
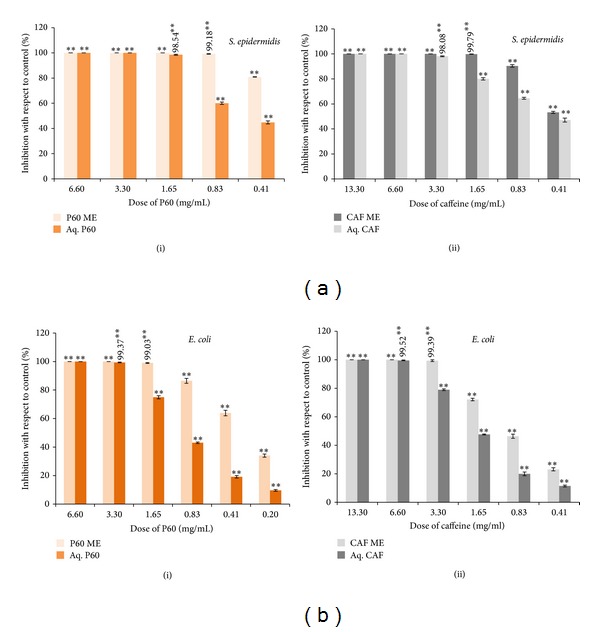
(a) Percentage inhibition of* S. epidermidis* by (i) P60 and its ME and (ii) Caffeine and its ME. Mean values of three independent experiments and S.E. are shown.  **Significant at *P* < 0.005. (b) Percentage inhibition of* E. coli* by (i) P60 and its ME and (ii) Caffeine and its ME. Mean values of three independent experiments and S.E. are shown.  **Significant at *P* < 0.005.

Minimum inhibitory concentration (MIC) is the lowest concentration of the agent that inhibits the turbid growth of the pathogen [17]. The results indicated that the growth of* S. epidermidis* and* E. coli* can be inhibited at a lower concentration when encapsulated in ME formulation compared to aqueous forms (Figures 3(a) and 3(b)). MIC values of aqueous P60, caffeine, and their MEs were estimated from percentage inhibition graphs and are shown in Table 3.

Combination of P60 and caffeine further lowered the MICs of individual agents in both aqueous form and ME formulation. However, FIC_index_ values for* S. epidermidis* were found to be correlated as synergistic in both aqueous form (FIC_index_ = 0.252) and ME formulation (FIC_index_ = 0.250) whereas for* E. coli* they are additive for aqueous form (FIC_index_ = 1.0) and synergistic for ME formulation (FIC_index_ = 0.5). This difference in the antibacterial spectrum of aqueous form (P60, caffeine, and P60 + CAF) and their ME formulations could be due to the basic difference in the morphology of gram-positive and gram-negative bacterial outer membrane. The outer membrane of gram-negative bacteria is composed of high content of lipids thereby less susceptible to any of the agents whilst preparation of MEs involves the use of surfactants that can effectively overcome the lipid barriers in gram-positive as well as gram-negative bacteria [25].

### 3.3. Free Radical Scavenging Activity

**Figure 4:**
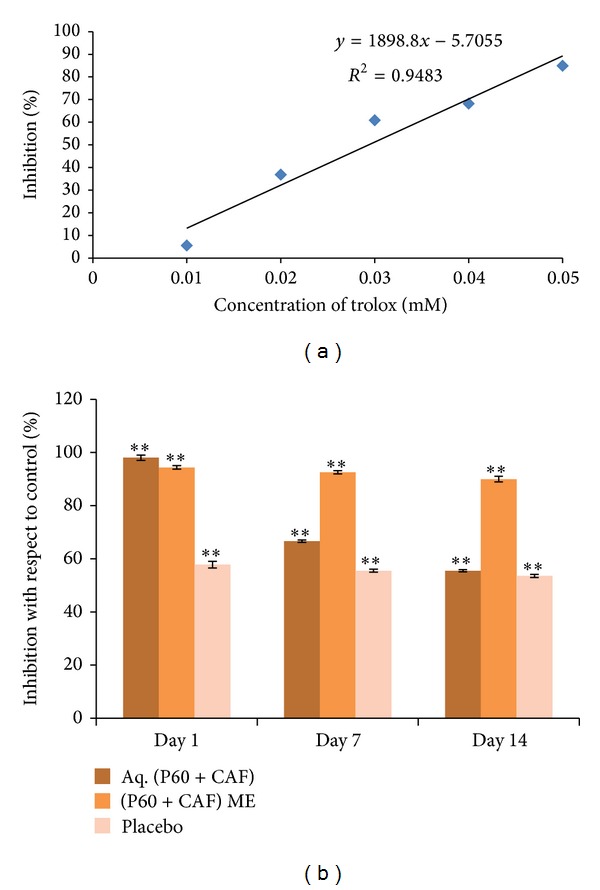
(a) Antioxidative effect of trolox (standard) using DPPH assay. (b) Antioxidative effect of aqueous P60 + CAF, its ME, and Placebo via DPPH assay. Data are represented as percentage of inhibition with respect to control. Mean values of three independent experiments and S.E. are shown.   **Significant at *P* < 0.005.

Based on the reported literature, bactericidal action of green tea catechins is due to hydrogen peroxide generated from the catechins. These catechins display strong antioxidant activity that efficiently eliminates reactive oxygen species. The antioxidant mechanism is believed to involve radical elimination by the phenolic hydroxyl group of the catechin structure. Trolox at different concentrations (0.005 to 0.05 mM) was used as a standard (Figure 4(a)).

Results indicated that ME formulation was effective as an antioxidative agent for at least up to two weeks as compared to the aqueous form (Figure 4(b)). This result was further confirmed by repeating the assay at 7th and 14th day and was in agreement with the work reported earlier. Arkawa et al. demonstrated that the hydrogen peroxide generation ability of EGCG was a central component with respect to bactericidal activity [26]. Green tea catechins play an important role in scavenging free radicals and while doing so they themselves might get oxidised. The encapsulation of active compound into a ME formulation sustained its antioxidative potential and functional stability [18]. Furthermore, it has been reported that green tea water extract was highly effective as a natural antioxidant for an oil-in-water emulsion storage [27].

### 3.4. Cytotoxicity Analysis on Vero Cell Lines

The presence of surfactants (high concentration) can cause irritation to normal mammalian cells when exposed directly [28]. However, nonionic surfactants (Cremophor EL and glycerol) as used in the present study have low toxicity compared to the cationic/anionic surfactants [29]. Therefore, to ascertain the safety profile of aqueous form and ME formulations of P60, caffeine, and P60 + CAF at their respective MIC values, the cytotoxicity analysis on Vero cell lines was carried out.

**Figure 5:**
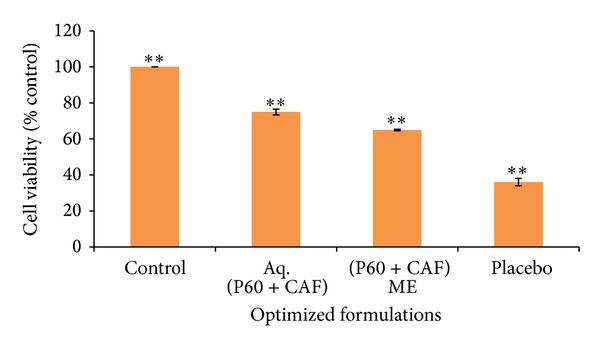
Cytotoxicity analysis of aqueous P60 + CAF, its ME, and corresponding placebo on Vero cell lines after 24 hrs via MTT assay. Data are represented as percentage of Vero cell viability. Mean values of three independent experiments and S.E. are shown.   **Significant at *P* < 0.005.

Results of MTT assay for cytotoxicity analysis of P60 + CAF in aqueous form and ME formulations are shown in the graph (Figure 5). Lower cell viability for ME (~65%) and placebo (~39%) compared to aqueous form (~75%) might be due to presence of surfactants that can be toxic to cells when present in high concentrations (in MEs) [30]. Therefore, P60 + CAF ME at its MIC value can be considered safe for usage and further recommended for* in vivo* studies.

## 4. Conclusion

Thermodynamically stable P60 + CAF loaded ME was developed and characterized by average particle size and TEM. Oil-in-water ME was found to be stable and of nanometric size. Antibacterial studies were carried out using disc diffusion assay and chequerboard microdilution assay. Lower MIC values of P60, caffeine, and P60 + CAF in ME formulations as compared to their aqueous forms indicated that MEs enhanced the antibacterial activity. This could be attributed to the formation of nanodroplets that disrupted bacterial cell wall nonspecifically. Furthermore, combination of P60 and caffeine in ME formulation was found to be synergistic against* E. coli*. Antioxidative DPPH assay showed that the radical scavenging potential of P60 + CAF ME is maintained up to 2 weeks compared to the aqueous combination and could be one of the mechanisms involved in bacterial cell death. Results of cytotoxicity analysis indicated that the concentration of P60 + CAF loaded ME at its MIC value is not cytotoxic to mammalian cells. The data pertaining to droplet size, PDI, zeta potential, zone of inhibition, MIC, antioxidative potential, and cytotoxicity studies was analyzed by one-way ANOVA and was found to be significant (at *P* < 0.05 and *P* < 0.005) as determined by Fisher (*F*) test *P* value calculator.

## Figures and Tables

**Table 1:** Droplet size, PDI, and zeta potential for P60 + CAF ME and placebo.

Formulation	Droplet Size (d*·*nm)	PDI	Zeta Potential (mv)
P60 + CAF ME	17.73** ± 0.12	0.229* ± 0.01	−10.43* ± 0.18
Placebo	12.78** ± 0.08	0.179* ± 0.01	−9.59* ± 0.19

Mean values of three independent experiments and S.E. are shown. ∗Significant at *P* < 0.05 and ∗∗significant at *P* < 0.005.

**Table 2:** Zone of inhibition (mm) of different formulations against *S. epidermidis* and *E. coli*.

	Aq. P60	P60 ME	Aq. CAF	CAF ME	Aq. (P60 + CAF)	(P60 + CAF) ME	Placebo
*S. epidermidis *	12.83 ± 0.44	15.00 ± 0.58	11.5 ± 0.29	14.17 ± 0.44	16.17 ± 0.17	18.83 ± 0.60	8.27 ± 0.15
*E. coli *	11.83 ± 0.44	13.67 ± 0.17	10.17 ± 0.20	11.00 ± 0.29	14.50 ± 0.29	16.50 ± 0.29	7.57 ± 0.30

Mean values of three independent experiments and S.E. are shown. Only statistically significant outcomes at *P* < 0.005 have been reported.

**Table 3:** MIC (mg/ml) of different formulations against *S. epidermidis* and *E. coli*.

	Aq. P60	P60 ME	Aq. CAF	CAF ME
*S. epidermidis *	1.63	0.83	3.30	1.65
*E. coli *	3.30	1.65	6.60	3.30

Only statistically significant outcomes at *P* < 0.005 have been reported.
